# Dizziness-related disability in persons with post-COVID condition: A cross sectional study

**DOI:** 10.3233/VES-230064

**Published:** 2024-08-29

**Authors:** Elin Östlind, Elisabeth Ekstrand, Iben Axén, Christina Brogårdh, Agneta Malmgren Fänge, Kjerstin Stigmar, Eva Ekvall Hansson

**Affiliations:** a Department of Health Sciences, Lund University, Lund, Sweden; b Dalby Healthcare Center, Dalby, Sweden; c Department of Hand Surgery, Skåne University Hospital, Malmö, Sweden; d Unit of Intervention and Implementation Research for Worker Health, Institute of Environmental Medicine, Karolinska Institutet, Stockholm, Sweden; e Department of Neurology, Rehabilitation Medicine, Memory Disorders and Geriatrics, Skåne University Hospital, Lund, Sweden; f Ear-Nose and Throat Department, Skåne University Hospital, Lund, Sweden

**Keywords:** Post-covid condition, dizziness, dizziness-related disability, activities of daily living

## Abstract

**BACKGROUND:**

Dizziness is a common symptom in post-COVID condition (PCC) which may have a large impact on several life domains. However, knowledge on dizziness-severity and disability in PCC is sparse.

**OBJECTIVE:**

The aim was to describe the severity of dizziness-related disability in individuals with PCC, and how it is manifested in daily life.

**METHODS:**

A questionnaire regarding symptoms of PCC, health, and dizziness-related handicap was administered online, and 524 persons with PCC and dizziness were included.

**RESULTS:**

Mean score of the Dizziness Handicap Inventory was 35.2 (24.0) and 51.8%, were classified as having moderate/severe dizziness-related disability. The percentage of maximum value for the subscales were: *Physical manifestation,* 48%, *Emotional Impact,* 36% and *Catastrophic Impact*, 17%. The greatest influence on physical movements was when bending forward, head shaking or doing strenuous physical activities or household chores.

**CONCLUSIONS:**

Half had moderate or severe dizziness-related disability and the physical manifestations occurred mostly during specific or strenuous body movements. This indicate a vestibular impairment that may be effectively managed with vestibular rehabilitation. Assessment and treatment of dizziness might be an essential part in PCC rehabilitation and future research should continue to explore the potential causal pathways of dizziness in PCC.

## 1 Introduction

The spread of Coronavirus Disease 2019 (COVID-19) continuously increases, with more than 750 million confirmed cases as of March 2023 [1]. Although a majority of infected individuals experience transient symptoms, some persist in having a variety of long-standing symptoms [2]. This condition has been clinically defined by the World Health Organization (WHO) that also proposed the name post COVID-19, now termed post-COVID condition (PCC) [3]. PCC is defined by the WHO as a condition that occurs in individuals with a probable or confirmed SARS-CoV-2 infection, with symptoms that last for at least 2 months and cannot be explained by an alternative diagnosis. A recent systematic review with meta-analysis showed a global PCC prevalence of 43% [4]. Symptoms such as fatigue, shortness of breath and cognitive dysfunction are common in PCC although there is a wide spectrum of symptoms from the cardiopulmonary, naso-oropharyngeal, musculoskeletal, and neuro-psychological systems [3–5]. Dizziness has also been reported as a symptom experienced by individuals with COVID-19 and PCC [6–8]. In persons with COVID-19 and PCC, dizziness often co-exist with other neurological manifestations such as headache, fatigue, and nausea [9]. The causal relationship between COVID-19 and dizziness is not fully understood although it seems to be related to factors such as secondary hypoxia, retrograde travel along the olfactory nerve and bulb, cytokine-related injury, and damage to specific receptors [10, 11]. Research has also suggested an association between PCC and autonomic dysfunction, postural orthostatic tachycardia syndrome (POTS) which, in turn, may cause dizziness [12].

Dizziness in the general population is relatively common with a reported life-time prevalence of 15–35% [13]. In general, dizziness can have a severe impact on function, wellbeing, work ability and health-related quality of life, and is associated with a considerable burden for the individual and the society at large [14, 15]. It is common among people with neurological conditions, but is also related to e.g., cardiovascular disorders and other diseases [16, 17]. These diseases and other comorbidities have also been shown to be a risk factor for poor COVID-19 outcome [18]. Effective treatments for dizziness are available, such as maneuver treatment for benign paroxysmal positional vertigo and vestibular rehabilitation for a wide range of diagnoses causing dizziness [19].

Overall, few studies on PCC and dizziness have been published and, to our knowledge, there are still no studies exploring the dizziness severity and the manifestations of dizziness-related disability for persons with PCC. Considering the vast number of people that may suffer from PCC and the impact that dizziness may have on several domains in life, it is important to gain more knowledge of the severity and manifestations of dizziness.

The aim of this study was to describe the severity of dizziness-related disability in individuals with post-COVID condition, and how it is manifested in daily life.

## 2 Methods

### 2.1 Study context and design

This survey study had a cross-sectional design and was part of a larger project (Life After Covid, LAC) investigating how PCC influence everyday life and perceived health. A previous study on consequences of PCC and factors associated with life satisfaction has been published [20].

### 2.2 Participants and recruitment

**Fig. 1:**
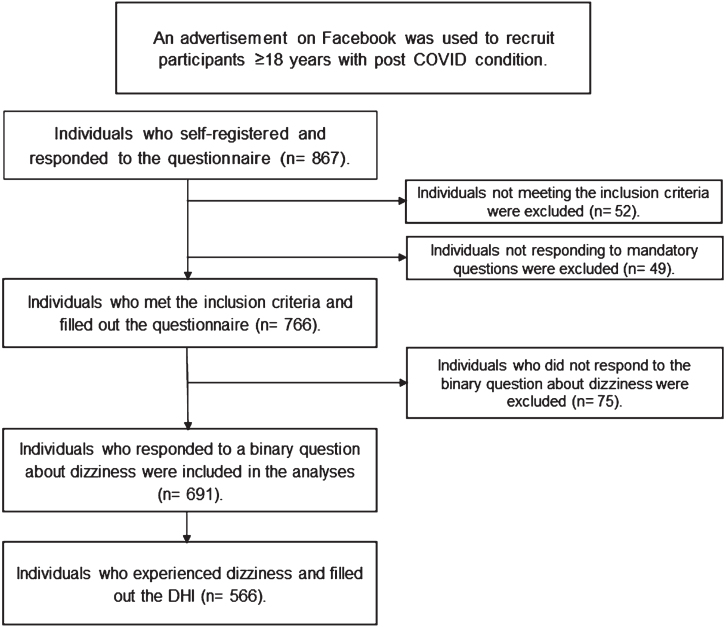
Flowchart of the recruitment process. a: Post COVID condition (PCC). b: Dizziness Handicap Inventory (DHI).

An advertisement on Facebook targeting persons living in the three most populated areas in Sweden was used to recruit participants to the project. The advertisement was hosted by Lund University and contained brief information about the project and its inclusion criteria. Those who were interested could follow a link to the project’s web page. The link to the web page was also shared on Twitter and Instagram. Inclusion criteria were age ≥18 years, having had COVID-19 and experiencing symptoms that had lasted for ≥2 months and being able to read and write Swedish. The project’s web page contained additional information, informed consent, and a link to an online survey. The advertisement on social media was active between the 21st of October and the 13th of November 2021, but the webpage and link to the survey were open until the 12th of February 2022. In total, 887 individuals responded to the survey but only participants that experienced disability due to dizziness were included in this study (*n* = 524). The recruitment process is illustrated in Fig. 1.

### 2.3 Data collection

Study data were collected from October 2021 and February 2022 and managed using Research Electronic Data Capture (REDCap) tools hosted at Lund University, Faculty of Medicine, Sweden [21, 22]. REDCap is a secure, web-based software platform designed to support data capture for research studies, providing 1) an intuitive interface for validated data capture; 2) audit trails for tracking data manipulation and export procedures; 3) automated export procedures for seamless data downloads to common statistical packages; and 4) procedures for data integration and interoperability with external sources.

### 2.4 Outcomes and measurements

#### 2.4.1 Sociodemographic data and post-COVID condition symptoms

Sociodemographic information regarding age, sex, family situation, education, comorbidities, and binary questions regarding several common PCC symptoms were collected in the survey.

#### 2.4.2 Dizziness

Dizziness was assessed through the binary question “Have you experienced dizziness at any time during the past year?”. This question has previously been used in other studies about dizziness prevalence [23].

Disability related to dizziness was measured with the *Dizziness Handicap Inventory* (DHI) [24]. DHI is a patient-reported outcome measure comprising 25 questions/items related to perceived disability due to dizziness in different situations and domains (work and leisure). Each item has three possible options, yes (4 points), sometimes (2 points) and no (0 points). All points are added up and result in a total score. Higher score indicates greater perceived disability caused by dizziness. There are three commonly used cut points in the DHI to assess the severity of the disability; mild (0–30 points), moderate (31–60 points) and severe (61–100 points) [25]. We calculated the total DHI score, and the proportion of participants categorized as mild, moderate, or severe dizziness-related disability.

In the original DHI, three content domains were developed to represent different aspects of the dizziness, namely *functional*, *physical,* and *emotional* subscales. However, in accordance with recommendations in previous research, these original subscales were not considered in this study [26]. Instead, newly developed subscales based on an exploratory factor analysis were used [27].

The three new subscales each consists of several specific items in the DHI: *Physical manifestations* (items 1, 5, 11, 13 and 25), *Emotional impact* (items 2, 22 and 23) and *Catastrophic impact* (items 9, 16, 20). To compare the results of the subscales, the mean score was divided with the total score of the subscale and presented as percentage of the total sum for each scale. The results for each DHI item were also calculated and presented as the proportion that answered yes/sometimes and no. The median (IQR) score for each item was calculated and presented.

DHI has shown sufficient construct validity and sufficient reliability for the total DHI score [26]. In this project, the Swedish translation of DHI by Jarlsäter and Mattsson was used [28]. Only individuals with a DHI score of ≥2 were included in the final analyses.

### 2.5 Data analysis

We used statistical package IBM SPSS Statistics version 27, (IBM Corp. Released 2020. Armonk, NY: IBM Corp). Total DHI score and subscale scores were presented as mean (standard deviation (SD)). The score of each DHI item was presented as median (IQR) and proportion answering yes, sometimes, or no, respectively.

## 3 Results

A total of 524 persons who had ongoing PCC and reported dizziness-related disability were included in the study. Their age range were 18–80 years. Participant characteristics are presented in Table 1. The most common PCC symptoms experienced by the participants were tiredness (88.7%), musculoskeletal pain (53.2%) and breathing difficulties (43.5%). A majority (71.4%) also experienced other symptoms, for example sore throat, nausea, and cognitive impairment.

**Table 1:** Sociodemographic characteristics of the participants (*n* = 524)

Characteristics
Age (years), mean (SD)	47.7 (10.4)
Sex, % (n)
Female	90.6 (475)
Male	7.8 (41)
Other/missing	1.5 (8)
Married or living with partner, % (n)
Yes	78.6 (412)
Postsecondary education, % (n)^a^
Yes	70.9 (370)
Source of income, % (n)^a^
Employment	63.7 (333)
Sickness benefit	22.2 (116)
Other sources of income	14.1 (74)
Other disease not related to COVID-19, % (n)^a^
Yes	42.4 (222)
Regular medication due to illness, % (n)^a^
Yes	50.0 (261)
Needed hospital care due to COVID-19, % (n)^a^
Yes	16.0 (83)
Duration of symptoms due to COVID-19 (months), mean (SD)	13.2 (5.1)
Participated in rehabilitation in relation to COVID-19, % (n)^a^
Yes	35.8 (187)

a: The results are presented as *valid* percent due to a few (1–4) missing responses.

### 3.1 Severity of dizziness-related disability

**Fig. 2:**
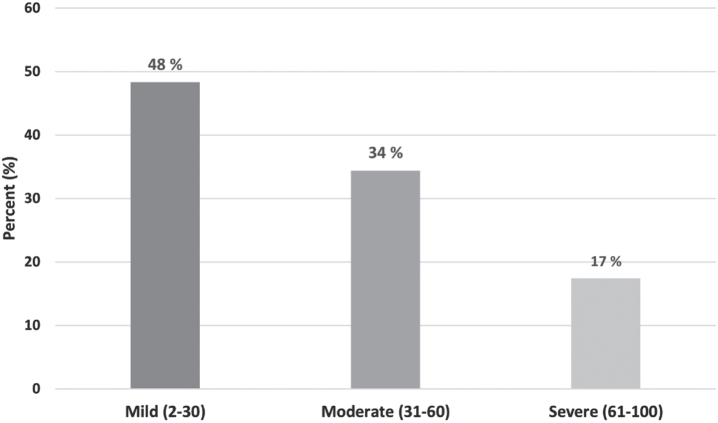
Classification of dizziness-related disability according to the Dizziness Handicap Inventory (DHI).

The mean (SD) score of the total DHI was 35.2 (24.0). A majority, 51.8%, were classified as having moderate or severe dizziness-related disability (Fig. 2).

### 3.2 Manifestations of dizziness-related disability in daily life

**Fig. 3:**
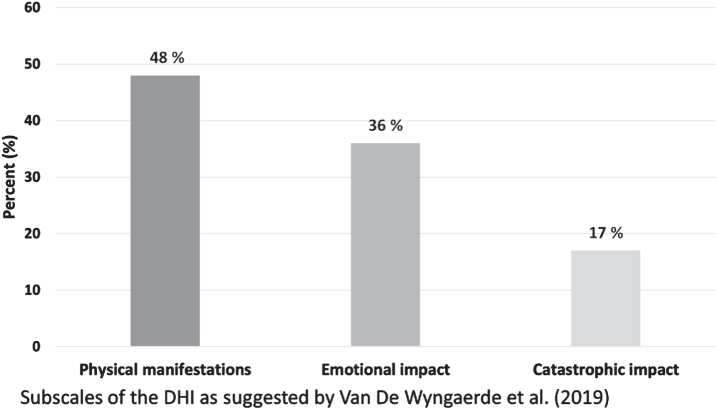
Mean value as percentage of total value for each subscale [27] of the Dizziness Handicap Inventory.

The mean (SD) values of the subscales were: *Physical manifestation*, 7.6 (4.5), *Emotional impact* 4.3 (3.7) and *Catastrophic impact*, 2.0 (3.1). In Fig. 3, the results of the subscales are compared as percentage of the maximum value of the subscale.

Several of the DHI items with the highest proportion answering *yes* were related to physical movement and more strenuous activities such as engaging in sports, doing household chores, quick movements of the head, bending forward or looking up. The participants also scored high on items related to feelings of frustration and items related to restriction of social activities, travelling and work. The median scores of all items and the proportion answering yes/sometimes/no are presented in Table 2.

**Table 2:** Items of the Dizziness Handicap Inventory

Item	Median	Response option (%)		
	(IQR)	No	Sometimes	Yes
1. Does looking up increase your problem? (P)	2 (0–4)	30	45	25
2. Because of your problem do you feel frustrated? (E)	2 (0–4)	28	31	41
3. Because of your problem do you restrict your travel for business or recreation?	2 (0–4)	40	25	35
4. Does walking down the aisle of a supermarket increase your problem?	0 (0–2)	55	27	18
5. Because of your problem do you have difficulty getting into or out of bed? (P)	0 (0–2)	61	30	9
6. Does your problem significantly restrict your participation in social activities, such as going out to dinner, going to the movies, dancing, or to parties?	2 (0–4)	42	25	33
7. Because of your problem do you have difficulty reading?	2 (0–2)	47	34	19
8. Does performing more ambitious activities like sports, dancing, household chores such as sweeping or putting dishes away, increase your problem?	2 (2–4)	22	36	42
9. Because of your problem are you afraid to leave your home without having someone accompany you	0 (0–2)	73	20	8
10. Because of your problem have you been embarrassed in front of others?	0 (0–2)	67	20	14
11. Do quick movements of your head increase your problem? (P)	2 (2–4)	20	32	48
12. Because of your problem do you avoid heights?	2 (0–4)	48	22	30
13. Does turning over in bed increase your problem? (P)	0 (0–2)	64	25	11
14. Because of your problem is it difficult for you to do strenuous housework or yard work?	2 (2–4)	33	38	29
15. Because of your problem are you afraid people may think that you are intoxicated?	0 (0–0)	77	13	10
16. Because of your problem, is it difficult for you to go for a walk by yourself? (C)	0 (0–2)	68	21	11
17. Does walking down a sidewalk increase your problem?	0 (0–2)	69	24	7
18. Because of your problem is it difficult for you to concentrate?	2 (0–2)	36	45	19
19. Because of your problem, is it difficult for you to walk around your house in the dark?	0 (0–2)	56	29	16
20. Because of your problem are you afraid to stay home alone? (C)	0 (0–0)	83	13	4
21. Because of your problem do you feel handicapped?	2 (0–2)	50	30	21
22. Has your problem placed stress on your relationships with members of your family and friends? (E)	0 (0–2)	61	22	17
23. Because of your problem are you depressed? (E)	0 (0–2)	63	26	11
24. Does your problem interfere with your job or household responsibilities?	2 (0–4)	38	33	28
25. Does bending over increase your problem? (P)	2 (0–4)	28	38	34

Each item had a response rate of >98%. Scoring: No: 0, Sometimes: 2, Yes: 4. P: Physical manifestations; E: Emotional impact; C: Catastrophic impact.

## 4 Discussion

In this sample of persons with PCC and perceived dizziness, a narrow majority were classified as having moderate or severe dizziness-related disability. The dizziness had an impact on several domains in their life and impaired their ability to participate in more strenuous physical activity, do household chores, work, and travel. High scores on items related to physical manifestations might implicate a vestibular impairment. The cause of the participants’ dizziness and dizziness-related disability is not known. The dizziness might be related to PCC, but it may also be related to other factors such as chronic diseases, medications, or musculoskeletal disorders. Comorbidity was common among the participants with 42% having another disease and 50% using medication due to illness. Compared to the general population, the participants in this study had been hospitalized at a higher rate due to COVID-19 [29] which is in line with previous studies reporting that illness severity during the acute phase and pre-existing comorbidities are associated with PCC [30]. Female sex is also a factor associated with PCC and in this study, a vast majority were women.

The pathophysiology for dizziness and other autonomic dysfunctions in PCC is largely still unknown although several potential mechanisms have been proposed [31]. As reported in previous research, dizziness seems to be a common symptom in both COVID-19 and PCC. A meta-analysis on neurological manifestations in COVID-19 found that dizziness had a pooled prevalence of 6.7% [9]. In PCC, a systematic review with meta-analysis on five studies showed a pooled dizziness prevalence of 26.4% [32] while a cross-sectional study reported a prevalence of dizziness as high as 58.5% [33]. In this study, we did not calculate the prevalence of present dizziness specifically but, nevertheless, most of the participants responding to the questionnaire did experience some kind of dizziness-related disability.

Only a few previous studies have used the DHI as an outcome measure in persons with COVID-19 or PCC. The mean total DHI score in this study, 35.2 is markedly higher than in a previous study including persons that had had COVID-19 [8]. That study showed a mean total DHI score of 16.1 but their participants were younger and had recovered from COVID-19. Another study using DHI also reported that dizziness-related disability was common in individuals with COVID-19 but that it decreased significantly during the months after the infection [34]. That study used different cut points on the DHI which makes comparison with our study difficult. In another study including participants with dizziness or balance complaints, 61.3$ had moderate to severe dizziness-related disability according to the DHI which is higher compared to the 51.8$ in this study [35]. However, all participants included in the previous study sought care for dizziness or balance problems which might explain the higher proportion of moderate and severe dizziness-related disability.

The DHI-items rated as most difficult by the participants were related to physical activity such as quick movements of the head, looking up or bending forward. There were also high scores on items related to more strenuous activities such as sports, household chores, engaging in social activities or travel. Based on the new subscales of the DHI, the results in this study indicate that the participants mainly had issues with physical manifestations related to dizziness [27]. They also scored high on item 2 that is related to the emotional subscale but this could represent an expression of sadness due to their dizziness-related problems. The overall lower scores on items related to the emotional and catastrophic subscales indicate that situations such as being home alone, walking alone or feelings of depression due to the dizziness are less prominent than activities and situations related to physical movements.

According to previous research, scoring high on DHI-items related to physical manifestations might indicate benign paroxysmal positional vertigo (BPPV) [36, 37]. BPPV is a common vestibular type of vertigo and may be effectively treated with maneuver treatment [38]. A wide range of other causes of dizziness is also effectively treated with vestibular rehabilitation [19]. Thus, once assessed and diagnosed, evidence-based and effective treatments for dizziness are available [39]. There are effective modalities for treating dizziness related to various diseases or disorders but the methods and effects of treating dizziness in a PCC population is still unknown. We suggest that future research focuses on conducting randomized controlled trials to explore if established and evidence-based treatments of dizziness could be effective in PCC rehabilitation as well.

### 4.1 Methodological considerations

A major strength in this study was the large sample size with >500 participants and that there were a wide age range. Another strength was that participation was not restricted by a geographically limited area. There were however some limitations that should be addressed. The binary question about dizziness captured any dizziness experienced during the past year and the dizziness might not have been explicitly related to COVID-19 or PCC. Furthermore, many participants had other diseases which may have caused the dizziness. We cannot say if there was a causal relationship between PCC and dizziness in our study.

The use of social media to recruit participants and online questionnaire to collect data are effective tools but they also have certain disadvantages. The recruitment method might have introduced a selection bias since there is a higher frequency of women engaged in social media [40]. Furthermore, the participants’ PCC diagnosis was self-reported and may thus be inaccurate. Some participants may also have found it difficult to distinguish disability due to dizziness with disability due to other PCC symptom since they manifest in similar ways.

The DHI classification mild (0–30)/moderate (31–60)/severe (61–100) developed by Whitney et al. [25] was used but in this study, we probably had some participants without present dizziness that responded to the DHI. Consequently, we chose to exclude participants that answered ‘No’ to all items and thus scored ‘0’ on the DHI. Furthermore, the original subscales in the DHI were opted out in favor of new subscales that was developed a few years ago in a re-assessment of the DHI [27]. The results from the subscales might thus be difficult to compare with previous research results using the original subscales. Nevertheless, we believe that using the new subscales was a strength since the they were developed in a more scientifically robust manner. Furthermore, presenting results of the DHI subscales and items provides a more in-depth knowledge of the domains and activities mostly affected by dizziness which might be useful information for researchers and clinicians.

## 5 Conclusions

This study provides important information on the severity of dizziness-related disability and a detailed description of what activities and situations that are most troublesome due to dizziness for persons with PCC. About half had moderate or severe dizziness-related disability, and physical manifestations involving bodily movements and more strenuous physical activities were mostly affected by the dizziness. These results indicate a vestibular impairment that could be effectively managed with vestibular rehabilitation. We suggest that future research continue to explore the potential causal pathways of dizziness in PCC and that persons with dizziness and PCC are assessed and treated in accordance with clinical guidelines.

## Funding

This research did not receive any specific grant from funding agencies in the public, commercial, or not-for-profit sectors.

## Ethical considerations

This study was performed in accordance with the WMA declaration of Helsinki. All participants gave their informed consent before answering the questionnaire by clicking on a link that directed them to the online survey. The study was approved by the Swedish Ethical Review Authority (Dnr 2020-02979).

## References

[1] WHO Coronavirus (COVID-19) Dashboard [Internet]. [cited 2022 Dec 13]. Available from: https://covid19.who.int

[2] Al-Aly Z. , Xie Y. and Bowe B. , High-dimensional characterization of post-acute sequelae of COVID-19, Nature 594(7862) (2021), 259–264. 33887749 10.1038/s41586-021-03553-9

[3] Soriano J.B. , Murthy S. , Marshall J.C. , Relan P. and Diaz J.V. , WHO Clinical Case Definition Working Group on Post-COVID-19 Condition. A clinical case definition of post-COVID-19 condition by a Delphi consensus, Lancet Infect Dis 22(4) (2022), e102–e107. 34951953 10.1016/S1473-3099(21)00703-9 PMC8691845

[4] Chen C. , Haupert S.R. , Zimmermann L. , Shi X. , Fritsche L.G. and Mukherjee. B. , Global Prevalence of Post-Coronavirus Disease (COVID-19]) Condition or Long COVID: A Meta-Analysis and Systematic Review, J Infect Dis 226(9) (2022), 1593–1607. 10.1093/infdis/jiac136 PMC9047189 35429399

[5] Aiyegbusi O.L. , Hughes S.E. , Turner G. , Rivera S.C. , McMullan C. , Chandan J.S. , et al., Symptoms, complications and management of long COVID: A review, J R Soc Med 114(9) (2021), 428–442. 34265229 10.1177/01410768211032850 PMC8450986

[6] Maleki M. , Maarefvand M. , Nazeri A.R. , Akbarzadeh Baghban A.R. and Borna A., Audio-Vestibular Profile of COVID-19; Systematic Review and Meta-analysis, Iran J Otorhinolaryngol 34(123) (2022), 145–155. 10.22038/IJORL.2022.60404.3079 PMC9393004 36035653

[7] Tabacof L. , Tosto-Mancuso J. , Wood J. , Cortes M. , Kontorovich A. , McCarthy D. , et al., Post-acute COVID-19 Syndrome Negatively Impacts Physical Function, Cognitive Function, Health-Related Quality of Life, and Participation, Am J Phys Med Rehabil 101(1) (2022), 48–52. 34686631 10.1097/PHM.0000000000001910 PMC8667685

[8] Yılmaz O. , Mutlu B.Ö. , Yaman H. , Bayazıt D. , Demirhan H. and Bayazıt Y.A. , Assessment of balance after recovery from Covid-19 disease, Auris Nasus Larynx 49(2) (2022), 291–298. 34503884 10.1016/j.anl.2021.08.011 PMC8405449

[9] Vitalakumar D. , Sharma A. , Kumar A. and Flora S.J.S. , Neurological Manifestations in COVID-19 Patients: A Meta-Analysis, ACS Chem Neurosci 12(15) (2021), 2776–2797. 34260855 10.1021/acschemneuro.1c00353

[10] Wu Y. , Xu X. , Chen Z. , Duan J. , Hashimoto K. , Yang L. , et al., Nervous system involvement after infection with COVID-19 and other coronaviruses, Brain Behav Immun 87 (2020), 18–22. 32240762 10.1016/j.bbi.2020.03.031 PMC7146689

[11] Desforges M. , Le Coupanec A. , Dubeau P., Bourgouin A., Lajoie L., Dubé M., et al., Human Coronaviruses and Other Respiratory Viruses: Underestimated Opportunistic Pathogens of the Central Nervous System? Viruses 12(1) (2020), 14. 10.3390/v12010014 PMC7020001 31861926

[12] Fedorowski A. and Sutton R. , Autonomic dysfunction and postural orthostatic tachycardia syndrome in post-acute COVID-19 syndrome, Nat Rev Cardiol 20(5) (2023), 281–282. 36732397 10.1038/s41569-023-00842-w PMC9893964

[13] Neuhauser H.K. . Chapter 5 – The epidemiology of dizziness and vertigo. In: Furman JM, Lempert T, editors. Handbook of Clinical Neurology [Internet]. Elsevier; 2016 [cited 2022 Dec 14]. p. 67–82. (Neuro-Otology; vol. 137). Available from: https://www.sciencedirect.com/science/article/pii/B9780444634375000054 10.1016/B978-0-444-63437-5.00005-4 27638063

[14] Neuhauser H.K. , Radtke A. , von Brevern M. , Lezius F., Feldmann M. and Lempert T., Burden of Dizziness and Vertigo in the Community, Arch Intern Med 168(19) (2008), 2118–24. 18955641 10.1001/archinte.168.19.2118

[15] Bronstein A.M. , Golding J.F. , Gresty M.A. , Mandalà M. , Nuti D. , Shetye A. , et al., The social impact of dizziness in London and Siena, J Neurol 257(2) (2010), 183–190. 19701661 10.1007/s00415-009-5287-z

[16] Kim H.A. , Ahn J. , Park H.S. , Lee S.M. , Choi S.Y. , Oh E.H. , et al., Cardiogenic vertigo: Characteristics and proposed diagnostic criteria, J Neurol 268(3) (2021), 1070–1075. 33025120 10.1007/s00415-020-10252-4

[17] Bösner S. , Schwarm S. , Grevenrath P. , Schmidt L. , Hörner K. , Beidatsch D. , et al., Prevalence, aetiologies and prognosis of the symptom dizziness in primary care – a systematic review, BMC Fam Pract 19(1) (2018), 33. 29458336 10.1186/s12875-017-0695-0 PMC5819275

[18] Treskova-Schwarzbach M. , Haas L. , Reda S. , Pilic A. , Borodova A. , Karimi K. , et al., Pre-existing health conditions and severe COVID-19 outcomes: An umbrella review approach and meta-analysis of global evidence, BMC Med 19(1) (2021), 212. 34446016 10.1186/s12916-021-02058-6 PMC8390115

[19] Han B.I. , Song H.S. and Kim J.S. , Vestibular rehabilitation therapy: Review of indications, mechanisms, and key exercises, J Clin Neurol Seoul Korea 7(4) (2011), 184–196. 10.3988/jcn.2011.7.4.184 PMC3259492 22259614

[20] Ekstrand E. , Brogårdh C. , Axen I. , Fänge A.M. , Stigmar K. and Hansson E.E. , Perceived Consequences of Post-COVID-19 and Factors Associated with Low Life Satisfaction, Int J Environ Res Public Health 19(22) (2022), 15309. 36430026 10.3390/ijerph192215309 PMC9690380

[21] Harris P.A. , Taylor R. , Thielke R. , Payne J. , Gonzalez N. and Conde J.G. , Research electronic data capture (REDCap)–a metadata-driven methodology and workflow process for providing translational research informatics support, J Biomed Inform 42(2) (2009), 377–381. 18929686 10.1016/j.jbi.2008.08.010 PMC2700030

[22] Harris P.A. , Taylor R. , Minor B.L. , Elliott V. , Fernandez M. , O’Neal L. , et al., The REDCap consortium: Building an international community of software platform partners, J Biomed Inform 95 (2019), 103208. 31078660 10.1016/j.jbi.2019.103208 PMC7254481

[23] Ekvall Hansson E. , Pessah-Rasmussen H., Bring A., Vahlberg B. and Persson L.: Vestibular rehabilitation for persons with stroke and concomitant dizziness-a pilot study, Pilot Feasibility Stud 6 (2020), 146. 33005434 10.1186/s40814-020-00690-2 PMC7526152

[24] Jacobson G.P. and Newman C.W. , The development of the Dizziness Handicap Inventory, Arch Otolaryngol Head Neck Surg 116(4) (1990), 424–427. 2317323 10.1001/archotol.1990.01870040046011

[25] Whitney S.L. , Wrisley D.M. , Brown K.E. and Furman J.M. , Is perception of handicap related to functional performance in persons with vestibular dysfunction? Otol Neurotol Off Publ Am Otol Soc Am Neurotol Soc Eur Acad Otol Neurotol 25(2) (2004), 139–143. 10.1097/00129492-200403000-00010 15021773

[26] Koppelaar-van Eijsden H.M. , Schermer T.R. and Bruintjes T.D., Measurement Properties of the Dizziness Handicap Inventory: A Systematic Review, Otol Neurotol 43(3) (2022), e282. 35147600 10.1097/MAO.0000000000003448

[27] Van De Wyngaerde K.M. , Lee M.K., Jacobson G.P., Pasupathy K., Romero-Brufau S., McCaslin D.L., The Component Structure of the Dizziness Handicap Inventory (DHI): A Reappraisal, Otol Neurotol 40(9) (2019), 1217. 31469793 10.1097/MAO.0000000000002365

[28] Jarlsäter S. and Mattsson E. , Test of reliability of the Dizziness Handicap Inventory and The Activities-specific Balance Confidence Scale for Use in Sweden, Adv Physiother 5(3) (2003), 137–144.

[29] Verity R. , Okell L.C. , Dorigatti I. , Winskill P. , Whittaker C. , Imai N. , et al., Estimates of the severity of coronavirus disease: A model-based analysis, Lancet Infect Dis 20(6) (2020), 669–677. 32240634 10.1016/S1473-3099(20)30243-7 PMC7158570

[30] Subramanian A. , Nirantharakumar K. , Hughes S. , Myles P. , Williams T. , Gokhale K.M. , et al., Symptoms and risk factors for long COVID in non-hospitalized adults, Nat Med 28(8) (2022), 1706–1714. 35879616 10.1038/s41591-022-01909-w PMC9388369

[31] Jammoul M. , Naddour J. , Madi A. , Reslan M.A. , Hatoum F. , Zeineddine J. , et al., Investigating the possible mechanisms of autonomic dysfunction post-COVID-19, Auton Neurosci Basic Clin 245 (2023), 103071. 10.1016/j.autneu.2022.103071 PMC9789535 36580747

[32] Pinzon R.T. , Wijaya V.O. , Jody A.A. , Nunsio P.N. and Buana: R.B. , Persistent neurological manifestations in long COVID-19 syndrome: A systematic review and meta-analysis, J Infect Public Health 15(8) (2022), 856–869. 35785594 10.1016/j.jiph.2022.06.013 PMC9221935

[33] Rodríguez-Pérez M.P. , Sánchez-Herrera-Baeza P. , Rodríguez-Ledo P. , Serrada-Tejeda S. , García-Bravo C. and Pérez-de-Heredia-Torres M. , Headaches and Dizziness as Disabling, Persistent Symptoms in Patients with Long COVID-A National Multicentre Study, J Clin Med 11(19) (2022), 5904. 36233769 10.3390/jcm11195904 PMC9572453

[34] Ludwig S. , Schell A. , Berkemann M. , Jungbauer F. , Zaubitzer L. , L, Huber, et al. Post-COVID-19 Impairment of the Senses of Smell, Taste, Hearing, and Balance, Viruses 14(5) (2022), 849. 35632590 10.3390/v14050849 PMC9145380

[35] Vanspauwen R. , Knoop A. , Camp S. , van Dinther J. , Erwin Offeciers F., Somers T., et al., Outcome evaluation of the dizziness handicap inventory in an outpatient vestibular clinic, J Vestib Res Equilib Orientat 26(5–6) (2016), 479–486. 10.3233/VES-160600 28262649

[36] Iglebekk W. and Tjell C. , High score of dizziness-handicap-inventory (DHI) in patients with chronic musculoskeletal pain makes a chronic vestibular disorder probable, Scand J Pain 22(3) (2022), 561–568. 35119799 10.1515/sjpain-2021-0102

[37] Chen W. , Shu L. , Wang Q. , Pan H. , Wu J. , Fang J. , et al., Validation of 5-item and 2-item questionnaires in Chinese version of Dizziness Handicap Inventory for screening objective benign paroxysmal positional vertigo, Neurol Sci Off J Ital Neurol Soc Ital Soc Clin Neurophysiol 37(8) (2016), 1241–1246. 10.1007/s10072-016-2573-2 27071688

[38] Power L. , Murray K. and Szmulewicz D.J. , Characteristics of assessment and treatment in Benign Paroxysmal Positional Vertigo (BPPV), J Vestib Res Equilib Orientat 30(1) (2020), 55–62. 10.3233/VES-190687 PMC9249279 31839619

[39] McDonnell M.N. , Hillier S.L. , Vestibular rehabilitation for unilateral peripheral vestibular dysfunction. Cochrane Database Syst Rev [Internet]. 2015 [cited 2023 Apr 3];(1). Available from: https://www.cochranelibrary.com/cdsr/doi/10.1002/14651858.CD005397.pub4/full?highlightAbstract=dizziness%7Cvertigo%7Cdizzi%7Cvertig 10.1002/14651858.CD005397.pub4 PMC11259236 25581507

[40] Whitaker C. , Stevelink S. and Fear N. , The Use of Facebook in Recruiting Participants for Health Research Purposes: A Systematic Review, J Med Internet Res 19(8) (2017), e290. 28851679 10.2196/jmir.7071 PMC5594255

